# Alveolar ridge augmentation using the shell technique with allogeneic and autogenous bone plates in a split‐mouth design—A retrospective case report from five patients

**DOI:** 10.1002/ccr3.3626

**Published:** 2020-12-29

**Authors:** Jochen Tunkel, Luca de Stavola, Anita Kloss‐Brandstätter

**Affiliations:** ^1^ private practice for oral surgery Bad Oeynhausen Germany; ^2^ private practice for oral surgery Rubano Italy; ^3^ Carinthia University of Applied Sciences Villach Austria

**Keywords:** allogeneic versus autogenous bone grafts, alveolar ridge augmentation, dental implants, guided bone regeneration, shell technique

## Abstract

Atrophic alveolar ridges of five patients were augmented with allografts and autografts on opposite sites, followed by dental implantation. Both augmentation materials led to equivalent bone gains. Allografts did not compromise the clinical outcome.

## Graphical abstract

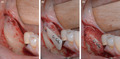

## 1 INTRODUCTION

Five patients with bilateral atrophy of the alveolar ridge were treated with allogeneic and autogenous augmentation on opposite sites, followed by dental implantation. Both augmentation materials led to equivalent horizontal and vertical bone gains. Thus, using allografts avoids bone block harvesting and does not compromise the patient's clinical outcome.

Tooth loss due is generally also associated with loss of bony structures. Hence, the consecutive insertion of an implant demands the more or less complex restoration of bony structures.^1^ Allogeneic or autogenous bone block transplantations or guided bone regeneration with bone granules and occlusive membranes demonstrated predictable and successful outcomes in alveolar ridge augmentation surgery.^2^, ^3^, ^4^

The “three‐dimensional” reconstruction or shell technique is a specific form of autogenous bone regeneration. Thin cortical bone blocks are initially used to restore the contours of the alveolar ridge and the resulting gaps are then filled with autogenous bone chips.^1^, ^5^ The resulting accelerated vascularization in the container and the greater volume stability of the avascular cortical bone plate reduces bone resorption to under 10%, and so the alveolar ridge contour can be restored with a predictable outcome.^6^, ^7^, ^8^ The low resorption rates even allow simultaneous insertion of implants in case of vertical bone augmentation.^9^ The short‐ and long‐term results after augmentation with the aid of the shell technique demonstrated low complication rates and excellent volume stability, even ten years after surgery.^10^

Alongside the use of the shell technique, there is also the possibility of reducing resorption processes by combining block transplantation with guided bone regeneration.^11^, ^12^ This method allowed resorption of autogenous monocortical bone blocks to be reduced to 5.5%‐7.2% between augmentation and implantation, and resorption over ten years to be reduced to only 0.8%.^11^, ^12^, ^13^ As a modification of this technique, the augmentation was performed with the shell technique and relining with xenogeneic granules, leading to a significantly lower rate of dehiscence and graft resorption.^8^ This “augmentative relining” method allowed the bone volume of the augmentation site to be increased by additional 17%, facilitated the incorporation of the bovine bone granulate in the regenerated bone and avoided further resorption until prosthetic treatment could be carried out.^8^

Nowadays, there are many instruments and options available to harvest intraoral bone.^14^ However, the majority of dentists working in implantology try to circumvent autogenous bone harvesting because of the limited amount of intraorally available bone. In recent years, xenogeneic, generally bovine materials have taken on an increasingly important role as substitutes for autogenous bone.^15^, ^16^

Allogeneic bone materials seem to be the closest available equivalents to autogenous bone transplants in clinical applications in terms of patient outcomes.^17^ As digitalization advanced, it became possible to mill allogeneic bone blocks to suit the defect geometry following preoperative diagnosis with cone beam computed tomography (CBCT) in order to insert these in a simplified surgical procedure.^3^, ^18^, ^19^ Allogeneic full block transplants are, however, subject to similar resorption processes as autogenous full block transplants.^4^ In a systematic overview, allogeneic transplants used for horizontal augmentation yielded similar gains as compared to intraorally harvested autogenous transplants.^20^ A recent study showed that the biomechanical properties of allogeneic cortical bone plates can be significantly improved by 10 min of rehydration, resulting in an increased breaking strength and flexibility.^21^ Thus, the use of cortical allogeneic bone plates, alike autogenous bone plates, could improve both the resorption and the complication rates and resolve the problem of insufficient intraoral bone quantity. The application of allogeneic bone blocks, however, has also been associated with complications such as incision line opening, perforations of the mucosa, dehiscences, mucosal irrigation, and infections leading in some cases to partial or total block loss.^22^, ^23^

A recent clinical study showed that there are no differences in complication rates and resorption rates between allogeneic and autogenic augmentation procedures of the alveolar ridge after 12 months,^4^ but there is still no scientific evidence for the equivalence of the two materials in their use in the more complex shell technique. In this study, radiological measurements of five patients with severely atrophic lower or upper jaws were retrospectively evaluated, where one side of the jaw was augmented with autogenous bone plates and the other side with allogeneic bone plates in a split‐mouth design.

This case series on five patients examined if an augmentation with allogeneic bone plates using the shell technique allows vertical and horizontal bone gains comparable to gains achieved with autogenous bone plates.

## 2 MATERIAL AND METHODS

### 2.1 Overview of the clinical cases

This case series is a retrospective examination of five patients who underwent surgery in a private practice in 2017‐2018. They all had a bilateral bone defect requiring vertical augmentation (three patients) or three‐dimensional horizontal augmentation in a buccal and oral orientation (two patients). The average width of the alveolar ridge was 3.7 ± 1.2 mm on the sides with a later allogeneic augmentation and on the sides with the later autogenous augmentation, the width of the alveolar crest was 3.9 ± 0.7 mm. The average height of the alveolar ridge was 3.0 ± 0.3 mm on the sides with a later allogeneic augmentation and on the side with the later autogenous augmentation, the width of the alveolar crest was 3.9 ± 1.3 mm. There were no statistically significant differences in the defect sizes, neither regarding the width of the alveolar ridge (Mann‐Whitney U test; *P* = .690), nor regarding the height of the alveolar ridge (Mann‐Whitney U test; *P* = .700).

The augmentations were performed using the shell technique. Due to the size of the defects, the augmentations could not be exclusively performed using the material available from a sole intraoral harvesting site. In two patients, a second retromolar bone harvesting procedure was not feasible, as this had already been carried out in previous years on one side and following consultation, the other three patients chose to avoid a second retromolar bone harvesting procedure. Instead, patients opted to rectify the bone deficit by allogeneic bone plates.

Four out of five patients had no general diseases that could affect the outcome of the implant treatment. One patient had an artificial heart valve that demanded prophylactic antibiotic treatment. Four out of five patients had periodontitis, three with stage II and one with stage I. All patients underwent non‐surgical treatment for periodontitis by the dentist, who had made the referral prior to implant treatment. The supportive periodontal therapy was then conducted at a four‐month interval. The patient without periodontitis had his teeth professionally cleaned in the week prior to surgery. In three patients, there was a free‐end situation in the lower jaw on both sides and in the upper jaw in two patients.

This study was a retrospective chart review, and it was in accordance with local institutional review board standards. All patients signed an informed agreement that their clinical parameters may be published.

### 2.2 Surgical procedure for alveolar ridge augmentation

All surgical procedures were carried out with intravenous sedation with midazolam (titrated between 9‐15 mg). Additionally, the patients were given metamizole sodium (1 g) iv intraoperatively. All patients received preoperative amoxicillin and clavulanic acid (875 mg/125 mg) iv during the augmentation and implantation. Systemic antibiotics were continued for ten days postoperatively with 875 mg/125 mg orally twice daily. Postoperative analgesia was provided with ibuprofen 400 mg prn. Patients were also instructed to rinse with ten ml chlorhexidine‐digluconate three times a day.

**Figure 1:**
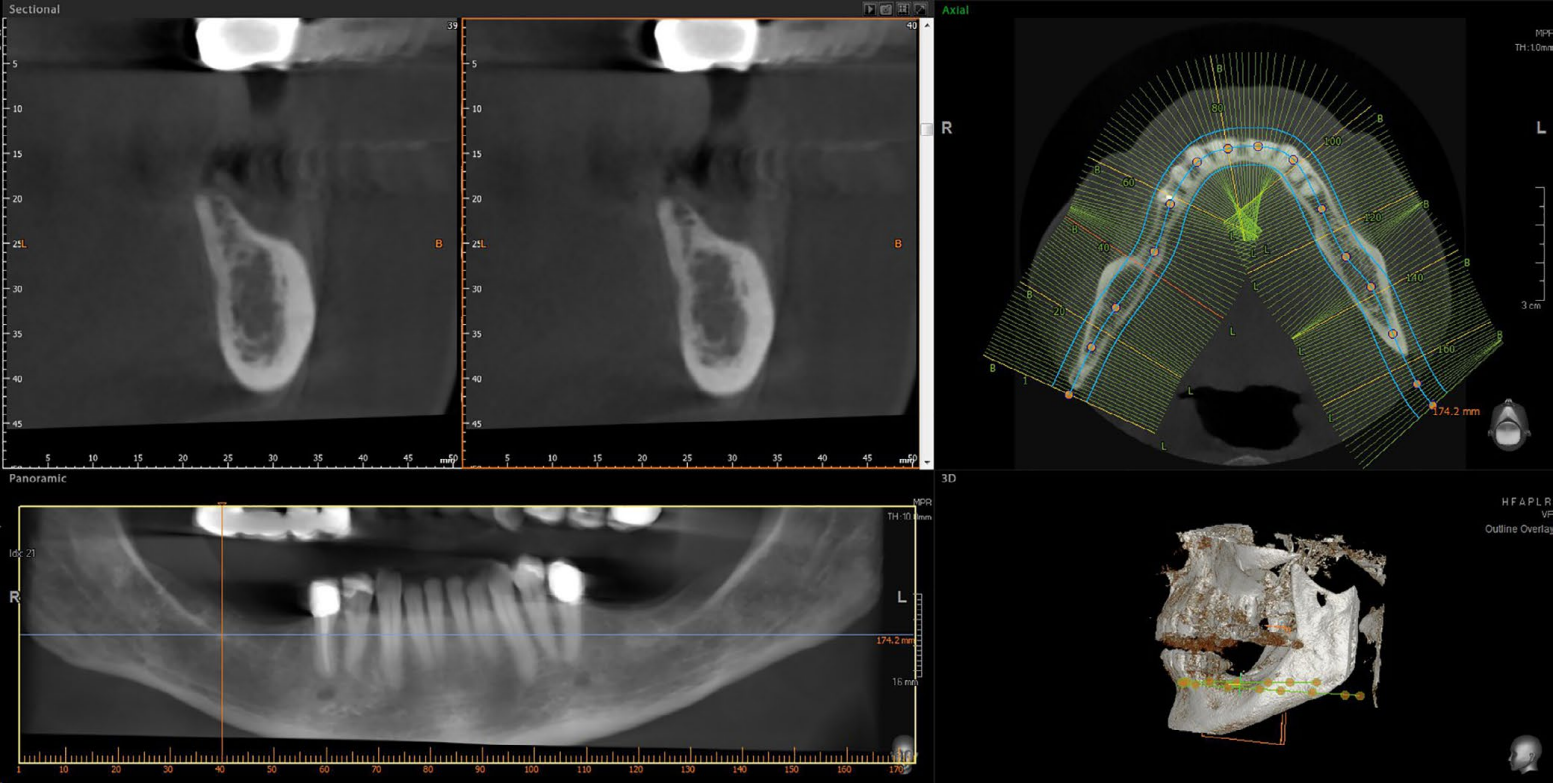
Preoperative CBCT: vertical bone defects in the third and fourth quadrant

The entire surgical procedure and dental restoration are showcased for the mandible of a female patient, aged 61 years, who exhibited signs of periodontitis (Figure 1).

**Figure 2:**
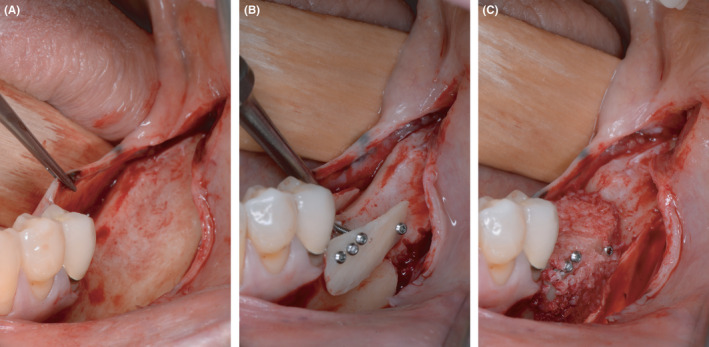
Augmentation in the third quadrant with autogenous bone plates and autogenous bone chips using the shell technique. A, horizontal and vertical defect in the third quadrant after flap elevation. B, buccal and lingual autologous bone plates fixed with microscrews utilizing the shell technique. C, bony envelope filled with autologous bone chips collected during thinning process of the bone plates

At the start of the procedure, a bone block was harvested from the left or right retromolar area using the Microsaw® (Dentsply Sirona Implants, Mannheim). This was then split lengthwise using a thin diamond disk. Subsequently, the plate was thinned with a Safescraper (Stoma, Tuttlingen) to a thickness of approximately 0.5 mm; autogenous bone chips were collected in the process. The plates obtained this way were secured buccally and lingually with four microscrews per plate (Microscrew®, Stoma, Tuttlingen). The bony envelope formed in this way was then filled with autogenous bone chips applied with slight pressure. Finally, blunt mobilization of the floor of the mouth was performed and a periosteal incision was made in the buccal region of the lower and upper jaw to allow tension‐free closure of the augmented area (Figure 2).

**Figure 3:**
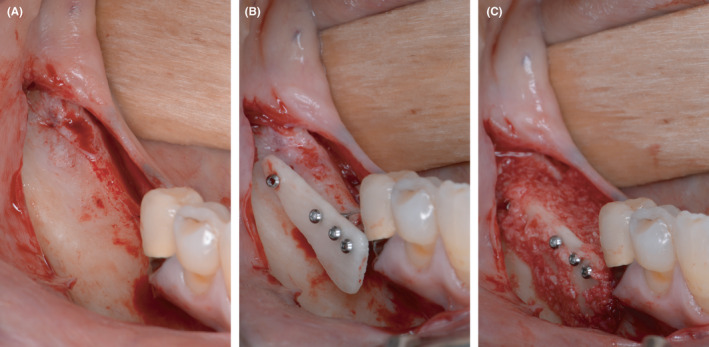
Augmentation in the fourth quadrant with allogeneic bone plates and autogenous bone chips using the shell technique. A, horizontal and vertical defect in the contralateral quadrant after flap elevation. B, buccal and lingual allogeneic bone plates fixed with microscrews utilizing the shell technique. C, bony envelope filled with autologous bone chips collected during thinning process of the autologous bone plates harvested in the third quadrant

Then, the augmentation in the contralateral quadrant was conducted. Here, an allogeneic bone plate (maxgraft® cortico, botiss biomaterials GmbH, Zossen, Germany) was initially immersed in sterile saline solution for 20 minutes. During this time, the flap was prepared. The allogeneic bone plate was divided based on the anatomical situation and secured buccally and orally with four microscrews. The resulting cavity was then filled with autogenous bone chips left retained from the augmentation in the contralateral quadrant. The augmentation site was closed using the same procedure as in the other quadrant (Figure 3).

### 2.3 Dental implantation

**Figure 4:**
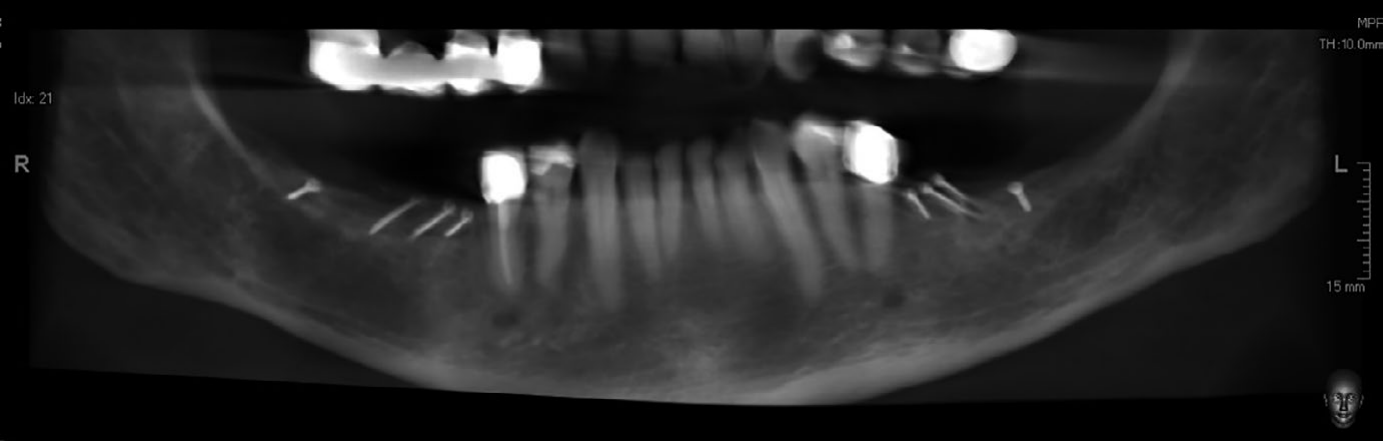
CBCT before implantation: significant vertical bone gain after 4 months of healing in both quadrants

**Figure 5:**
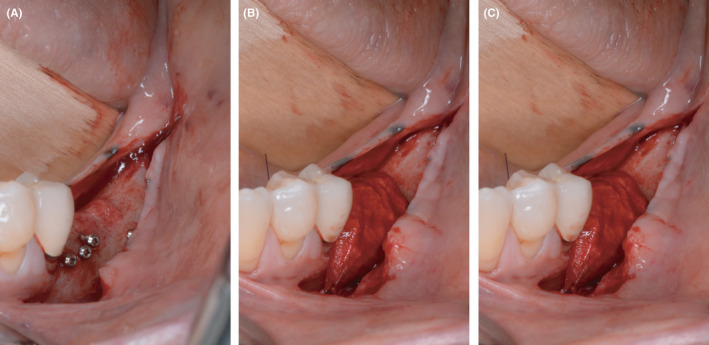
Implantation in the third quadrant and augmentative relining with bovine bone substitute material and collagen membrane.A, regenerated bone of the autologous bone plate site with only minor signs of resorption. B, situation after insertion of two bone level implants and fixation of a collagen membrane for the relining GBR. C, relining process finished with DBBM particles and membrane stabilized by resorbable sutures

**Figure 6:**
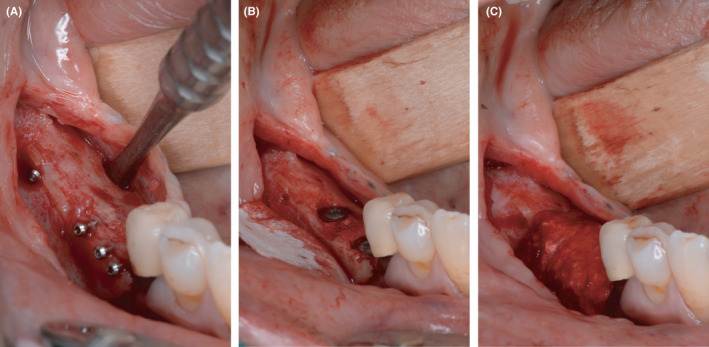
Implantation in the fourth quadrant and augmentative relining with bovine bone substitution material and collagen membrane. A, regenerated bone in the allogeneic bone plate site with only minor signs of resorption, as well. B, situation after insertion of two bone level implants and fixation of a collagen membrane for the relining GBR. C, relining process finished with DBBM particles and membrane stabilized by resorbable sutures

**Figure 7:**
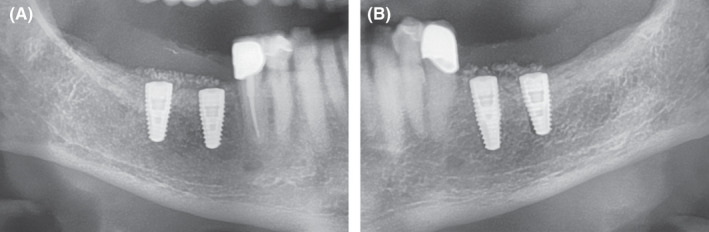
Extracts from postoperative OPG after implantation and GBR in the third and fourth quadrant. A, fourth quadrant. B, third quadrant

After a healing phase of four to five months, the reentry was performed in both quadrants. Before implantation, a CBCT was taken (Figure 4). Following a crestal incision and flap mobilization, the inserted microscrews were removed from both sides. Every patient received in total four implants, with two implants being inserted in each augmentation side. Bone level tapered implants with SLActive surface (Straumann GmbH, Freiburg, Germany) were inserted according to the manufacturer's instructions (Figure 5 for implantation after autogenous augmentation and Figure 6 for implantation after allogeneic augmentation). Sufficient amounts of bone tissue were present with about 1 to 2 mm of bone on the buccal and lingual / palatal wall distant from the implant. Following buccal incision of the periosteum, a collagen membrane (Jason® membrane, botiss biomaterials GmbH, Zossen, Germany) was attached to the apical periosteum with resorbable sutures. The section of the alveolar ridge was then covered with bovine bone material (cerabone^®^, botiss biomaterials GmbH, Zossen, Germany) with the same layer thickness as the particle size (1.0‐2.0 mm) and the membrane was secured with resorbable sutures on the oral side of the flap. This augmentative relining was then covered the same way as applied for the shell technique. Figure 7 shows extracts from postoperative OPG after implantation and GBR in the third and fourth quadrant.

### 2.4 Exposure of implants

**Figure 8:**
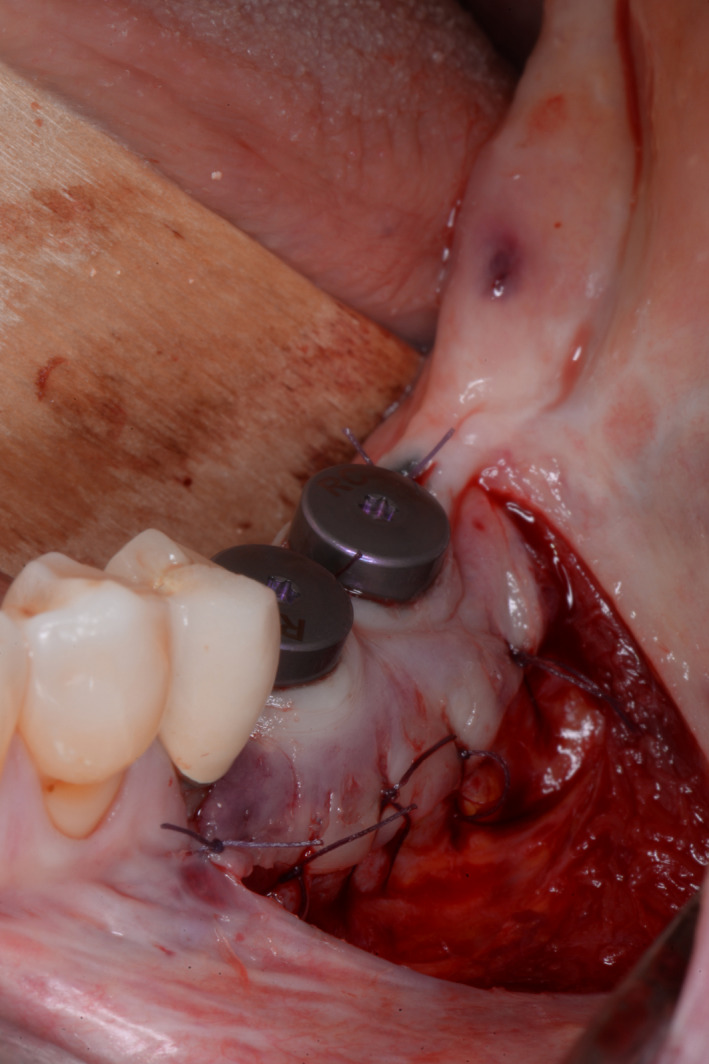
Exposure of the implants in the third quadrant by stab incision combined with vestibuloplasty according to Kazanjian

**Figure 9:**
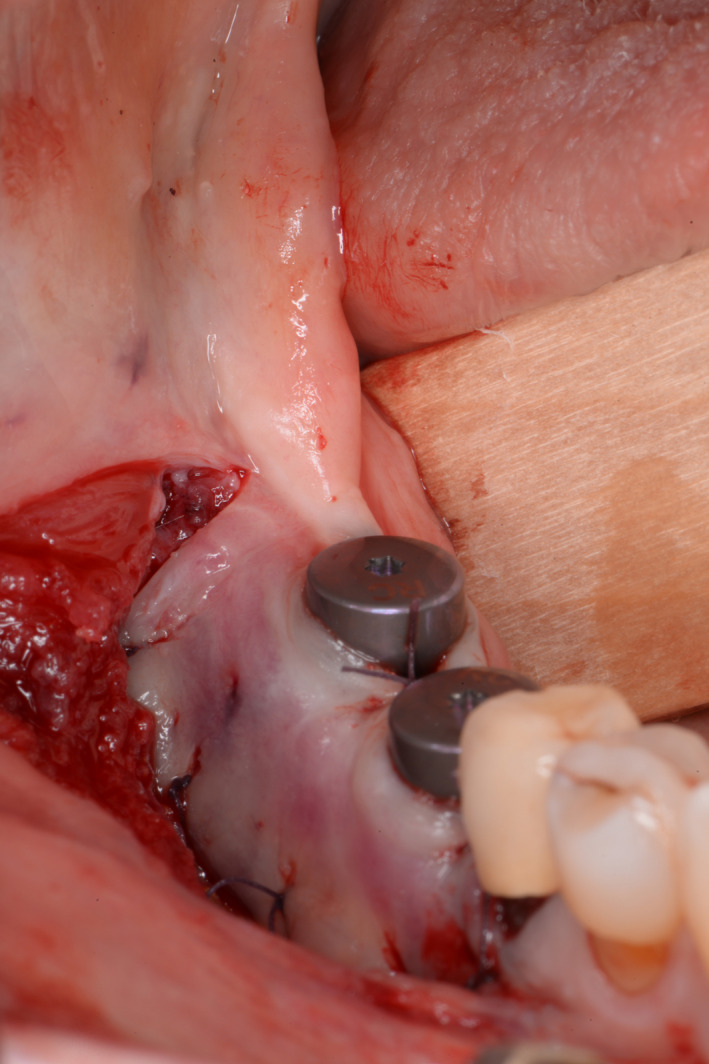
Exposure of the implants in the fourth quadrant by stab incision combined with vestibuloplasty according to Kazanjian

**Figure 10:**
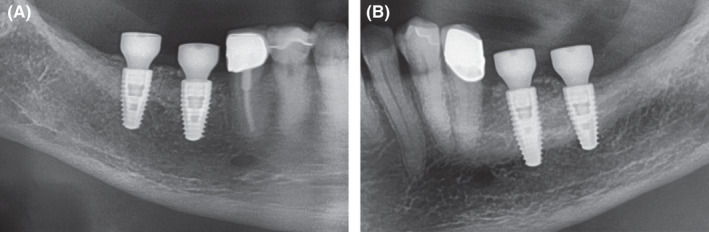
Extracts from postoperative OPG: Gingival former in the third and fourth quadrants in situ. A, Allogeneic site showing good integration of the implants and no loss of crestal bone. Relining layer of DBBM particles in situ. B, Same situation on the autologous site showing similar results compared to the allogeneic site

**Figure 11:**
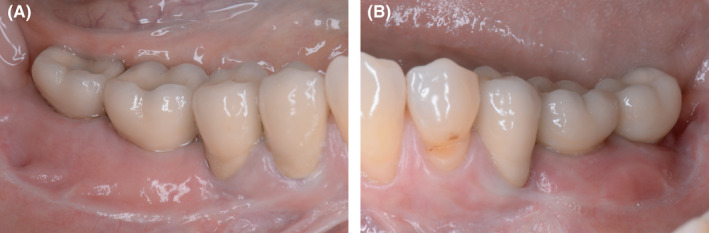
Clinical situation after prosthetic treatment in the third and fourth quadrant. A, Clinical result on the allogeneic site after prosthetics. Fixed mucosa was increased by Kazanjian vestibuloplasty to 5‐6mm. B, Similar clinical situation on the autologous bone plates site

**Figure 12:**
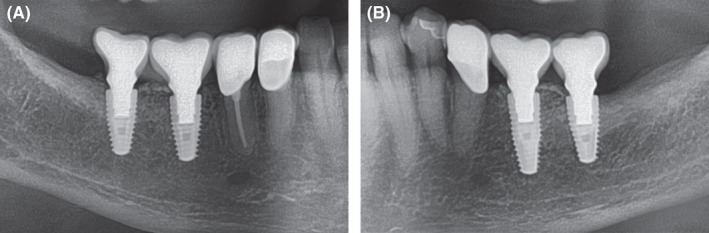
Extracts from OPG: Radiological situation after prosthetic treatment. A, Allogeneic site with good integration of the implants and no crestal bone loss and well‐integrated relining layer. B, Autologous site showing good integration, no bone loss and well‐integrated relining layer on the autologous site

After a healing time of 5 months, the implants were exposed. As the area had been augmented twice, there was a lack of keratinized tissue in the region of implants. For this reason, a vestibuloplasty according to Kazanjian was performed in the lower jaw and an apically shifted mucosal flap procedure was performed in the upper jaw.^24^, ^25^, ^26^, ^27^ In the mandible, this involved sharp separation of the muscle from the periosteum in an apical direction after initial preparation of a supra‐muscular mucosal flap. The mucosal flap was secured to the periosteum with resorbable sutures. The implants were exposed by stab incision (Figure 8 for implantation after autogenous augmentation and Figure 9 for implantation after allogeneic augmentation). Conical gingival formers (Conical Shape^®^, Straumann GmbH, Freiburg, Germany) with a diameter of 6.5 mm were used as healing abutments. In the maxilla, a two‐layer mucosal flap was prepared starting at an incision line displaced in a palatal direction that allowed a sufficient band of keratinized mucosa to be shifted apically. After insertion of the same conical gingival former as in the lower jaw, the flap was fixed to the buccal periosteum with sutures (Figure 10). After a healing time of six weeks, the dentist who made the referral performed the prosthetic treatment (Figure 11). Figure 12 depicts the radiological situation after prosthetic treatment.

### 2.5 Measuring alveolar ridge changes

Every patient was subjected to three‐dimensional x‐ray diagnostics (CBCT), followed by computer‐aided planning of the augmentation and subsequent implantation. In total, two CBCTs were recorded for each patient, one before treatment and one directly before implantation. At each time point, the alveolar bone levels were measured in their height, width, and depth at the planned site of the mesial implant, at the site of the distal implant and in the center of the two planned positions.

At the other time points, the measurements were taken with a reference template and a caliper. After exposure of the bone, the horizontal width of the alveolar ridge was measured at two positions with a caliper: at the planned site of the mesial implant and at the site of the distal implant. As the bone could no longer be fully exposed during second stage surgery, the thickness of the bone and mucosal soft tissue was measured. A probe was then used to measure the thickness of the mucosa buccally and orally, and this measurement was deducted from the measurement taken with the caliper. The values were rounded to the closest half millimeter.

Preoperative vertical volume tomography was used to measure the depth of the vertical defect. This involved drawing a horizontal line at the planned augmentation height and measuring the distance of the alveolar ridge at the planned implantation site and the midpoint to this line.

The drilling template in the form of a miniplast splint, which was perforated at the two measuring points, was used to measure the vertical changes. The first measurement was taken directly after the augmentation. The vertical changes were then measured by subtracting the measuring results received during implant surgery.

### 2.6 Statistical analyses

Statistical analyses were performed with IBM SPSS (version 27; International Business Machines Corp., Armonk, NY, USA). For descriptive statistics, mean values and standard deviations were calculated. For pairwise comparisons, non‐parametric Mann‐Whitney U tests were applied for testing of the null hypothesis that the distribution of two groups (eg, horizontal bone gain after allogeneic versus autogenous augmentation) was equal.

## 3 RESULTS

All surgical procedures healed without complications in each patient, both on the allogeneic and autogenous augmentation sites. The follow‐up time was 12 months.

The five patients were on average 60.8 years old (Table 1). Four patients were females. Only one patient did not suffer from periodontitis. All patients were non‐smokers.

**Table 1:** Basic characteristics of the patients

	Patient 1	Patient 2	Patient 3	Patient 4	Patient 5
Gender	male	female	female	female	female
Age	65	61	61	60	57
Comorbidities	none	Heart valve replacement	none	none	none
Periodontal status	Periodontitis stage I grade B	Periodontitis stage II grade B	Periodontitis stage I grade B	Periodontitis stage II grade B	Periodontitis stage II grade B
Periodontitis adequately treated	yes	yes	yes	yes	yes
Oral hygiene	good	good	sufficient	good	sufficient
Bleeding on probing	<15%	<15%	<25%	<15%	<25%
Complications	Temporary sensitivity disturbance on the donor side	none	none	none	none
Treated jaw	lower	lower	lower	upper	upper
Allogeneic augmentation on	right side	right side	right side	left side	right side

### 3.1 Horizontal gain and resorption

The average horizontal gain was 5.8 ± 0.4 mm on the autogenous side and 6.1 ± 0.6 mm on the allogeneic side (Table 2), with no difference in the horizontal gain between autogenous and allogeneic sides (Mann‐Whitney U test; *P* = 0,310). In the four months to implantation, an average of 0.3 ± 0.3 mm (5%) was lost on the autogenous side and 0.1 ± 0.1 mm (2%) on the allogeneic side (Table 2). Due to the augmentative relining carried out at the implantation, there was an additional horizontal bone gain of 1.0 ± 0.2 mm (14%) on average between augmentation and exposure on the autogenous site and 0.8 ± 0.2 mm (11%) on the allogeneic site (Table 2). At implantation, there were no differences in the horizontal bone gain and resorption rates between the autogenous and allogeneic sides (Mann‐Whitney U test; *P* = .841).

**Table 2:** Changes to the horizontal width of the alveolar ridge on the surgical side treated with *autogenous* or *allogeneic* bone plates and autogenous bone chips

Patient Nr, augmentation material	Width of alveolar ridge before augmentation	Horizontal gain after augmentation	Horizontal loss (shrinkage) after 4 months	Horizontal gain after relining at implantation	Width of alveolar ridge after 12 month
1, allogeneic	5.3 mm	+5.2 mm	−0.2 mm	+0.5 mm	10.8 mm
1, autogenous	4.8 mm	+5.3 mm	−0.2 mm	+0.8 mm	10.8 mm
2, allogeneic	4.2 mm	+6.0 mm	−0.0 mm	+0.8 mm	11.0 mm
2, autogenous	4.0 mm	+6.0 mm	−0.5 mm	+1.0 mm	10.5 mm
3, allogeneic	3.8 mm	+6.5 mm	−0.3 mm	+1.0 mm	11.0 mm
3, autogenous	4.3 mm	+5.7 mm	−0.0 mm	+1.2 mm	11.2 mm
4, allogeneic	2.8 mm	+6.2 mm	−0.0 mm	+1.0 mm	10.0 mm
4, autogenous	3.3 mm	+6.2 mm	−0.7 mm	+1.0 mm	9.8 mm
5, allogeneic	2.2 mm	+6.8 mm	−0.0 mm	+0.8 mm	9.8 mm
5, autogenous	3.0 mm	+6.0 mm	−0.2 mm	+1.2 mm	10.0 mm

Note

The values indicated are mean values obtained from three measuring sites.

### 3.2 Vertical gain and resorption

In three patients, also a vertical augmentation had to be carried out. The average vertical gain was 3.9 ± 1.3 mm on the autogenous site and 3.2 ± 0.3 mm on the allogeneic site (Table 3), with no difference in the vertical gain between autogenous and allogeneic sides (Mann‐Whitney U test; *P* = .700). In the period until implantation, an average of 0.3 ± 0.0 mm (8%) was lost on the autogenous site and 0.3 ± 0.3 mm (9%) on the allogeneic site (Table 3). Due to the augmentative relining carried out at the implantation, there was an additional bone gain between augmentation and exposure averaging 0.3 ± 0.3 mm (8%) on the autogenous and 0.5 ± 0.5 mm (16%) on the allogeneic site (Table 3). Again, there were no differences in the vertical bone gain and resorption rates between the autogenous and allogeneic sides (Mann‐Whitney U test; *P* = .700).

**Table 3:** Changes to the vertical height of the alveolar ridge on the surgical side treated with *autogenous* bone plates and *autogenous* bone chips

Patient Nr, augmentation material	Vertical defect of the alveolar ridge before augmentation	Vertical gain after augmentation	Vertical loss (shrinkage) after 4 months	Vertical gain after relining at implantation	Vertical bone gain after 12 months
1, allogeneic	3.5 mm	+3.5 mm	−0.3 mm	+0.0 mm	3.2 mm
1, autogenous	5.3 mm	+5.3 mm	−0.3 mm	+0.0 mm	5.0 mm
2, allogeneic	3.0 mm	+3.0 mm	−0.5 mm	+1.0 mm	3.5 mm
2, autogenous	3.8 mm	+3.8 mm	−0.3 mm	+0.5 mm	4 mm
3, allogeneic	3.2 mm	+3.2 mm	−0.0 mm	+0.5 mm	3.7 mm
3, autogenous	2.7 mm	+2.7 mm	−0.3 mm	+0.5 mm	2.9 mm

Note

The values indicated are mean values obtained from three measuring sites.

## 4 DISCUSSION

In this clinical case series, bilateral vertical and horizontal defects were augmented in the right and left upper and lower jaw using autogenous and allogeneic bone plates. With the use of allogeneic bone plates, a second retromolar bone block harvesting was avoided and thus the operative trauma for the patient was reduced. Additionally, this retains the option of using the untouched retromolar donor area for later augmentations.

Autogenous bone transplants are generally accepted to be the gold standard in augmentative surgery, especially in vertical augmentations.^28^, ^29^ This is also attributed to the low rate of complications.^2^ The most serious problems with autogenous full block transplants reported in the literature are the resorption rates of 21%‐25% along with the restricted availability.^10^, ^12^, ^29^, ^30^, ^31^, ^32^ The shell technique according to Khoury was developed to circumvent this problem.^1^, ^5^ On the one hand, the shell technique allows efficient use of the bone block so that a much larger bone volume can be augmented with the same harvesting volume, and on the other hand, the resorption rates with this technique are reduced to 5%‐9% by obeying the principles of biological healing.^8^, ^33^, ^34^ Vertical bone augmentations generally and the shell technique specifically are technically sophisticated methods that demand superior surgical skills.^18^ The risk of complications involved in bone harvesting, especially nerve damage, remain, however, a major follow‐up clinical trial conducted by experienced surgeons demonstrated this risk to be marginal.^10^, ^35^

With the application of allogeneic bone blocks intraoral or even extraoral bone harvesting can be avoided, reducing the overall risk of complications and patient morbidity.^36^, ^37^ The clinical results are partially comparable with autogenous transplants.^4^ There is also the opportunity to significantly reduce operating times when using CAD/CAM milled allogeneic bone blocks.^18^, ^38^, ^39^, ^40^ However, depending on the size of the reconstruction and the structure of the transplant, allogenic bone blocks are subject to comparable resorption processes as autogenous bone blocks, whereas vertical bone resorption might even be more pronounced.^11^, ^12^, ^20^, ^29^, ^30^, ^31^

Positive experiences with allogeneic bone blocks are indicated for a wide range of bony defects including horizontal and vertical alveolar ridge augmentations,^41^, ^42^, ^43^ post‐traumatic reconstruction,^44^, ^45^ and sinus lift with reduced residual bone height.^46^, ^47^ Even cleft lip and palate have been successfully treated with allogeneic blocks.^38^, ^48^ The most common complications associated with allogenic bone blocks were membrane exposure and loosening of the osteosynthesis screws.^49^

While the use of allogeneic particulate material and blocks in different variations has been published, there is little information on the use of allogeneic cortical plates for the shell technique.^50^, ^51^ The combination of allogeneic bone plates with autogenous bone chips used in this case series appears to be a promising alternative to autogenous transplants in complex bone augmentation procedures. Firstly, the risk of complications associated with bone harvesting is avoided, as only bone chips have to be collected with the aid of a bone scraper. Depending on the required volume, this can cause slight morbidity, while the risk of nerve lesions can be widely eliminated. Secondly, the available bone substance can generally be increased without restriction, so that there is enough material for augmentation in all four quadrants. This case series suggests that the resorption behavior of allogeneic and autogenous bone plates is comparable. Thus, the alveolar process can be restored with predictable outcomes without the need for over‐augmentation, as a resorption rate of 5%‐9% can be assumed.^8^, ^33^ Even in the event of resorption of the thin allogeneic bone plate after implantation, the implants would not be exposed, as they were inserted in the area of the autogenous bone due to the shell technique. Reduced osseointegration is therefore not expected. Another step when working with allogeneic bone plates would be to completely avoid the harvesting of autogenous bone. This would require the use of allogeneic bone chips. Generally, the use of a barrier membrane to cover the augmented area is still recommended when solely applying allogenic materials. It can be assumed that there would be an increased complication rate in the form of dehiscence resulting from membrane application.^11^, ^12^, ^52^, ^53^, ^54^ This combination of allogeneic bone plates with autogenous bone chips therefore appears to be an excellent compromise to reduce donor site morbidity and the risk of complications without compromising the outcome, even in the case of vertical augmentations.

The additional use of augmentative relining and over‐augmentation with xenogeneic bone substitute material and collagen membrane is intended to avoid bone resorption in the period between implantation and prosthetic treatment, especially in the first 12 months after augmentation when the bone is subject to continuous restructuring.^8^ Combining autogenous bone plates with the delayed relining technique led to an additional increase of the bone volume.^8^ The relining technique used could decrease bone loss.

The question arises why one side was grafted with autogenous bone while the other side was grafted with an allogeneic cortical plate in combination with autogenous bone chips. When treating all five patients, the first intention was to graft both sides with autologous bone. At the time of treatment, allogeneic bone plates were quite new in the market and the authors did not consider them being a standard augmentation procedure. In two patients, a bone harvesting in one retromolar area had already been done before, and therefore, not enough bone could be harvested from one retromolar area. Therefore, it was decided in accordance with the patient to harvest autologous bone at one retromolar side and complement the missing bone volume by allogeneic bone plates. In three cases, the patients wanted to reduce the morbidity of the procedure. As there was a strong intention to fill the gap in between the plates by autologous bone chips, it was decided again in accordance with the patient that one bone harvesting was obligatory and the other could be avoided using allogeneic bone plates.

Allogeneic bone chips were not used in any of the five patients, as the authors were convinced at that time point that the gap had to be filled with autologous bone chips. The delayed guided bone reconstruction with xenogeneic bone particles and collagen membrane was first described by de Stavola and Tunkel in 2013.^8^ The aim of this technique was to have a second layer of a material that is slowly or better not resorbed. This layer's function was to protect the underlying autologous (or allogeneic) bone during the two‐year remodeling phase and prevent an early periimplant bone loss.

Our case series suffers from several limitations. The sample size of five patients limits the generalizability of the results. The detection of no difference between two augmentation materials does not imply that there is no difference at the population level, as the sample size was too low and therefore lacked statistical power. However, the fact that the two augmentation materials (allogeneic and autogenous) were used within the same patients reduced the possibility of other potential biases such as inadequate sample selection. In addition, vertical augmentations were only performed in three patients, thereby further reducing the sample size. Still, the observation of a vertical bone gain of more than 3 mm with both materials underscored the possibility to reconstruct massive defects in the alveolar crest without the need of bone harvesting from extraoral sites.

This case series demonstrates that the augmentative relining technique can also be carried out with allogeneic bone plates. No clinical problems were observed in association with this procedure, and there were signs of good integration of the xenogeneic bone substitute into the augmented bone. Even after a prolonged restructuring time for allogeneic bone plates, the augmentative relining offered protection from undesirable postoperative bone loss in the long term.

### 4.1 Summary

This clinical case series emphasized that by means of the shell technique equal horizontal and vertical bone gain with both autogenous and allogeneic bone plates is achievable. The additional implementation of augmentative relining with a xenogeneic bone substitute material and collagen membrane seemed to minimize resorption processes and to maintain bone volume in the long term. However, this observation needs further replication in larger studies including a control group without relining.

## CONFLICT OF INTEREST

JT receives honoraria for lectures for the companies Botiss and Straumann, the manufacturer and the provider of the bone plates. There are no conflicts of interest with the results of the publication. The other authors declare no conflicts of interest.

## AUTHOR CONTRIBUTIONS

Jochen Tunkel: designed the study; treated the patients and performed surgical procedures; was involved in drafting the manuscript; performed literature research; analyzed the data; gave final approval of the version to be published. Luca de Stavola: was involved in drafting the manuscript; made substantial contributions to conception and design; gave final approval of the version to be published. Anita Kloss‐Brandstätter: was involved in drafting the manuscript; made substantial contributions to evaluation and statistics; gave final approval of the version to be published.

## ETHICS STATEMENT

This study was a retrospective chart review and it was in accordance with local institutional review board standards. All patients signed an informed agreement that their clinical parameters may be published.

## Data Availability

All data collected for this study are summarized in Tables 1, 2, and 3. The single measurements per patient can be obtained upon request by Jochen Tunkel.
